# Gut Microbiota and Ischemic Stroke: From Pre‐Stroke Dysbiosis and Acute‐Phase Changes to Therapeutic Applications

**DOI:** 10.1002/brb3.71547

**Published:** 2026-08-15

**Authors:** Ruihui Weng, Gaolei Dong, Jianxin Tang, Yu Zhao

**Affiliations:** ^1^ Department of Neurology, National Clinical Research Center For Infectious Diseases The Third People's Hospital of Shenzhen Shenzhen China; ^2^ Department of Liver Surgery & Liver Transplantation Center, National Clinical Research Center For Infectious Diseases The Third People's Hospital of Shenzhen Shenzhen China

**Keywords:** gut‐brain axis communication, gut dysbiosis, ischemic stroke, stroke outcomes, therapeutic strategies

## Abstract

**Purpose:**

Ischemic stroke poses a major global health burden, and growing evidence points to the microbiota–gut–brain axis as an important contributor to stroke pathophysiology and recovery. The purpose of this review is to summarize current knowledge on bidirectional gut‐brain communication in stroke, establishing gut dysbiosis as an upstream risk factor for stroke onset and examining microbiota dynamics across acute and chronic phases.

**Method:**

A comprehensive review of the current literature was conducted to synthesize existing evidence on the mechanisms of gut‐brain communication, the influence of host factors, post‐stroke complications, and candidate therapeutic interventions targeting the MGBA in ischemic stroke.

**Finding:**

Gut dysbiosis acts as a risk factor for stroke through TMAO‐mediated atherothrombosis, systemic inflammatory priming, and comorbidity‐associated dysbiotic pre‐conditioning. Following a stroke, dysbiosis is characterized by a depletion of short‐chain fatty acid (SCFA)‐producing taxa and an expansion of pro‐inflammatory bacteria, particularly *Proteobacteria* and *Enterobacteriaceae*. Host factors (age, sex, genetics, and comorbidities) shape microbiota signatures and influence outcomes. Furthermore, post‐stroke complications—including cognitive impairment, depression, stroke‐associated pneumonia, and gastrointestinal dysfunction—are intimately linked to persistent dysbiosis. Mechanistically, the gut microbiota modulates stroke outcomes through immune regulation, metabolite signaling, blood–brain barrier integrity, and vagal pathways. Candidate therapeutic interventions (probiotics/prebiotics, fecal microbiota transplantation, SCFA supplementation, traditional Chinese medicine, and neuromodulation) demonstrate promising neuroprotective and recovery‐enhancing effects, predominantly in preclinical models.

**Conclusion:**

While targeted interventions like probiotics and dietary fiber supplementation have shown modest benefits in small clinical trials, human clinical evidence remains largely preliminary. The translation of FMT, SCFA supplementation, and neuromodulatory approaches to clinical practice requires validation through rigorous, adequately powered randomized controlled trials. Future precision microbiome medicine demands individualized profiling and phenotype‐specific interventions to establish microbiota‐targeted strategies as viable adjunctive stroke therapies.

## Graphical abstract

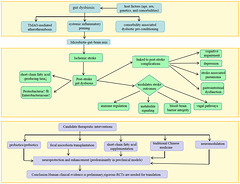

**Abbreviations**
AhR: aryl hydrocarbon receptor
AIS: acute ischemic stroke
ASBT: apical sodium‐dependent bile acid transporter
BBB: blood–brain barrier
BCCAO: bilateral common carotid artery occlusion
CE: cardioembolic
CRP: C‐reactive protein
CS: cryptogenic stroke
ENS: enteric nervous system
FMT: fecal microbiota transplantation
FUT2: fucosyltransferase 2
GI: gastrointestinal
GPR41/43: G protein‐coupled receptor 41/43
HDAC: histone deacetylase
HPA: hypothalamic‐pituitary‐adrenal
HQLDG: Huang‐Qi‐Long‐Dan‐Gen particles
HTPR: high on‐treatment platelet reactivity
iFABP: intestinal fatty acid binding protein
IL: interleukin
IPA: indole‐3‐propionic acid
IS: ischemic stroke
LAA: large artery atherosclerotic
LBP: LPS‐binding protein
LCI: lacunar cerebral infarction
LPS: lipopolysaccharide
MCAO: middle cerebral artery occlusion
MGBA: microbiota–gut–brain axis
NAFLD: non‐alcoholic fatty liver disease
NF‐κB: nuclear factor kappa B
NG‐R1: notoginsenoside R1
NIHSS: National Institutes of Health Stroke Scale
OSAHS: obstructive sleep apnea hypopnea syndrome
PAGln: phenylacetylglutamine
PAMPs: pathogen‐associated molecular patterns
PLR‐RS: pueraria lobata root‐resistant starch
PSCCID: PSCI with comorbid depression
PSCI: post‐stroke cognitive impairment
PSD: post‐stroke depression
SAP: stroke‐associated pneumonia
SCFA: short‐chain fatty acid
T2D: type 2 diabetes
tDCS: transcranial direct current stimulation
TGR5: Takeda G protein‐coupled receptor 5
TLR4: toll‐like receptor 4
TMA: trimethylamine
TMAO: trimethylamine N‐oxide
TNF‐α: tumor necrosis factor alpha
Treg: regulatory T cell
UDCA: ursodeoxycholic acid
VNS: vagus nerve stimulation

## 1 Introduction

Ischemic stroke (IS) is a leading cause of morbidity and mortality worldwide, posing a significant burden on healthcare systems and society (Hilkens et al. 2024). Recent research has demonstrated that the gut microbiota, serving as the “second genome” of the human body, represents a complex microbial ecosystem. It plays crucial roles in various physiological processes, including digestion, immune regulation, and neurological function (Fan and Pedersen 2021). Dysbiosis, the imbalance of gut microbiota, has been increasingly recognized as a contributing factor to stroke risk and severity, as well as a potential therapeutic target (Koszewicz et al. 2021). Critically, gut microbiota dysbiosis operates not merely as a post‐stroke consequence but as a bona fide upstream risk factor for IS onset. Accumulating epidemiological and mechanistic evidence demonstrates that pre‐existing microbial imbalances—characterized by enrichment of trimethylamine N‐oxide (TMAO)‐producing taxa (e.g., *Prevotella* and *Escherichia/Shigella*), depletion of short‐chain fatty acid (SCFA)‐producing genera (e.g., *Roseburia* and *Faecalibacterium prausnitzii*), and expansion of pro‐inflammatory *Proteobacteria*—are strongly associated with atherosclerosis, platelet hyperreactivity, systemic endotoxemia, and neuroinflammatory priming, thereby increasing cerebrovascular vulnerability (Tang et al. 2013; Yin et al. 2015; Nemet et al. 2020). Comorbidities highly prevalent in stroke patients—including hypertension, type 2 diabetes (T2D), obesity, and metabolic syndrome—are independently associated with characteristic pre‐stroke dysbiotic signatures that further amplify ischemic risk (J. Li et al. 2017; Iatcu et al. 2021; H. Wang et al. 2021; dos Santos and Galie 2024). This aligns with the recently proposed “gut‐brain‐vascular axis” framework, which integrates neurovascular integrity, microbial metabolites, and systemic inflammation into a unified model of stroke pathogenesis (Hendel et al. 2026). This review therefore adopts a comprehensive perspective, examining gut microbiota both as an upstream risk determinant and as a dynamic mediator across the acute and chronic phases of IS, ultimately informing microbiota‐targeted therapeutic strategies.

Microbiota–gut–brain axis (MGBA), the bidirectional communication between the gut and brain, plays a pivotal role in linking gut microbiota to IS (Honarpisheh et al. 2022). Through the MGBA, gut signals reach the brain via systemic inflammation and metabolites, neural pathways, and immune circuits; reciprocally, brain injury reshapes gut physiology and ecology. Clinical and experimental studies link gut microbiota and gut‐derived metabolites (SCFAs, TMAO, tryptophan catabolites, and bile acids) (Peh et al. 2022) to atherosclerosis, thrombosis, neuroinflammation, and recovery trajectories (J. Lee et al. 2020). Interventions (dietary fiber, pre/probiotics/synbiotics, FMT, targeted metabolites, traditional Chinese medicine (TCM), and neuromodulation) show promise but require phenotype‐specific deployment and rigorous trials.

This review aims to provide a comprehensive overview of the mechanistic and clinical evidence across phases of stroke, and integrate comorbidities and host factors. By synthesizing recent findings, we highlight the potential of gut microbiota as a novel target for stroke prevention and treatment, offering insights into future research directions and clinical applications.

## 2 Literature Search Strategy

To ensure transparency and reproducibility, we describe herein the methodology used to identify the literature included in this review. A systematic search of the following electronic databases was conducted: PubMed/MEDLINE, Embase, Web of Science, and the Cochrane Library. The search covered publications from January 2013 to January 2026. Given the rapidly evolving nature of the field, we prioritized studies published within this period; however, seminal and foundational studies published prior to 2015 were selectively included when they provided essential mechanistic context or represented landmark contributions that could not be omitted (e.g., pivotal studies on TMAO and atherothrombosis and foundational germ‐free animal experiments). The following Medical Subject Headings (MeSH) terms and free‐text keywords were used in combination: “ischemic stroke” OR “cerebral ischemia” OR “acute ischemic stroke”; AND “gut microbiota” OR “gut microbiome” OR “intestinal microbiota” OR “gut dysbiosis”; AND related terms including “microbiota‐gut‐brain axis,” “short‐chain fatty acids,” “TMAO,” “trimethylamine N‐oxide,” “fecal microbiota transplantation,” “probiotics,” “prebiotics,” “blood‐brain barrier,” “neuroinflammation,” “post‐stroke cognitive impairment,” “post‐stroke depression,” “stroke‐associated pneumonia,” and “comorbidities.” Searches were limited to articles published in English. Titles and abstracts of identified records were screened for relevance by two independent reviewers (R.W. and G.D.), with full‐text articles retrieved for studies meeting the inclusion criteria. Reference lists of retrieved articles were additionally hand‐searched for any relevant studies not captured by the database search. Reviews, meta‐analyses, randomized controlled trials, observational studies, and experimental animal studies were all considered eligible for inclusion. No formal PRISMA flow diagram was generated, as this is a narrative review rather than a systematic review; nonetheless, we have endeavored to achieve comprehensive and balanced coverage of the available evidence.

## 3 MGBA

**FIGURE 1:**
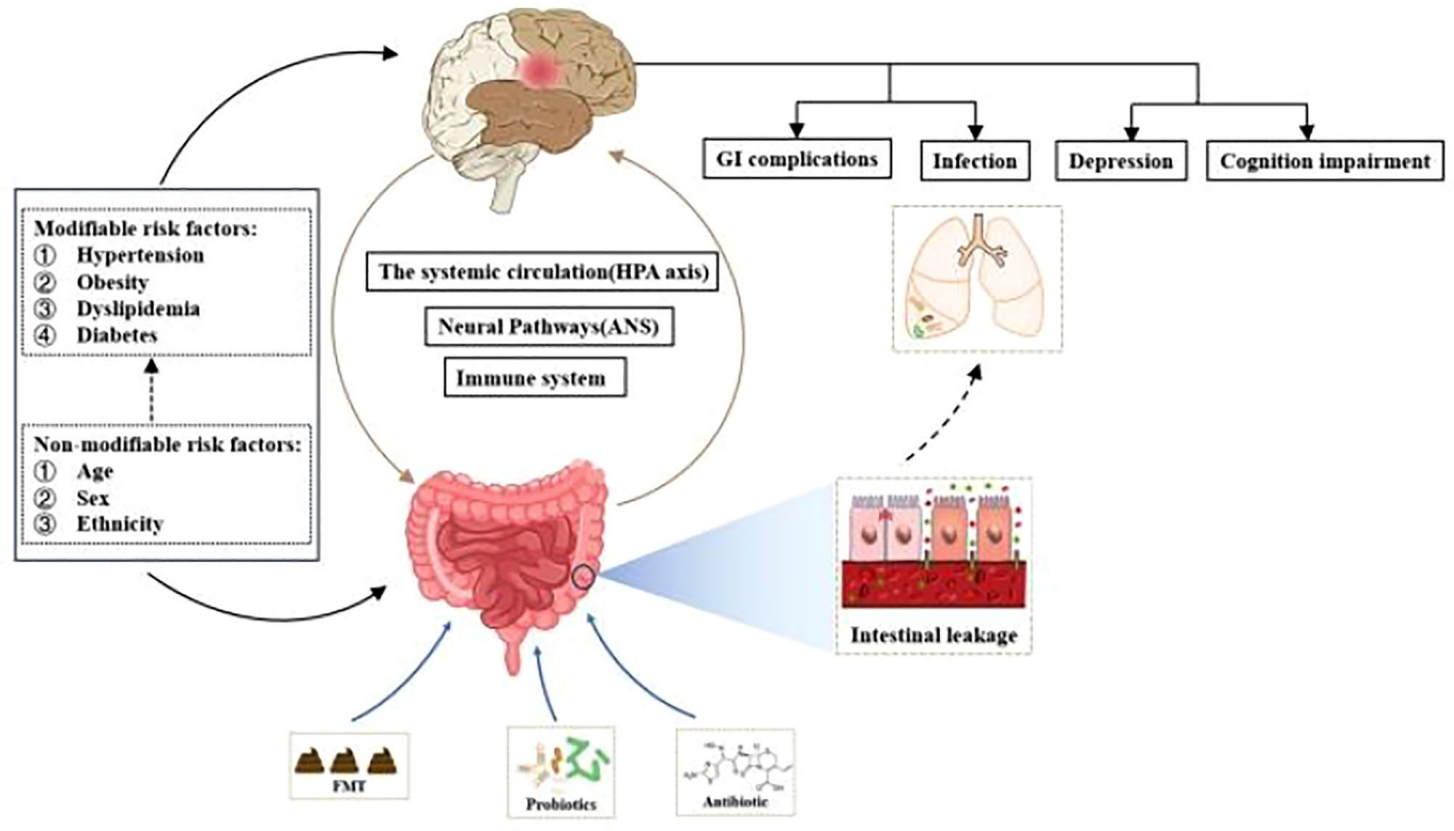
The bidirectional communication pathways between the gut microbiota and brain. The gut microbiota could bidirectionally communicate with the brain through multiple pathways, including the systemic circulation (HPA axis), neural pathways (ANS), and immune system. Modifiable risk factors and non‐modifiable risk factor not only participate in the development and occurrence of stroke, but also affect the gastrointestinal microbiome. When stroke happens, it causes gut microbiota dysbiosis, decreases mucus and goblet cells production, reduces gut motility, as well as increases gut permeability. Thus, gastrointestinal microbiome could translocate to organs, like lung, which could increases infection and death. Moreover, gut microbiota dysbiosis is associated with complications and the severity of stroke and worse prognosis. Fecal microbiota transplantation, probiotics, and antibiotic might be beneficial for prevention and treatment of stroke.

The MGBA operates through four interconnected pathways that collectively regulate brain‐gut homeostasis and respond dynamically to cerebral ischemia (Figure 1).

### 3.1 Systemic Inflammatory and Metabolic Circulation

Within the systemic inflammatory and metabolic circulation arm of the microbiota–gut–brain axis, endocrine‐stress signaling via the hypothalamic–pituitary–adrenal (HPA) axis calibrates immune tone and epithelial barrier function, while cortisol surges and sympathetic activation reshape microbiota composition and increase intestinal permeability (W. Zhang, Dong, et al. 2023; S. Y. Zhou et al. 2023). Concomitantly, stroke‐associated dysbiosis is linked to elevated circulating IL‐6 (where IL is interleukin), tumor necrosis factor alpha [TNF‐α], IL‐1β, and lipopolysaccharide (LPS), perturbing the regulatory T cell [Treg]/Th17/γδT balance and facilitating meningeal T‐cell trafficking that amplifies neuroinflammation (Benakis et al. 2016; Xu et al. 2021). At the metabolite level, SCFAs sustain intestinal and blood‐brain barrier integrity and promote microglial homeostasis and Treg induction (J. Lee et al. 2020; Zhu et al. 2021), whereas TMAO enhances atherothrombosis and platelet reactivity (Yin et al. 2015); shifts in tryptophan metabolism toward kynurenine, together with altered serotonin signaling, further modulate neuroimmune circuits (Gao et al. 2020; Y. Huang et al. 2023), and phenylacetylglutamine (PAGln) links aromatic amino acid catabolism to cardiovascular and neuroinflammatory risk (Nemet et al. 2020). Collectively, the combination of elevated LPS and TMAO with depletion of SCFAs establishes a pro‐thromboinflammatory milieu and compromises gut and BBB barriers, mechanistically predisposing patients with acute ischemic stroke (AIS) to larger infarcts and a higher burden of complications.

### 3.2 Neural Pathways

The bidirectional communication between the gut microbiota and central nervous system in IS operates through three principal neural pathways. The vagus nerve serves as the primary conduit, transmitting afferent signals from enteroendocrine cells to the nucleus tractus solitarius while modulating gut motility and immune responses via efferent projections (J. Zhang, Ling, et al. 2024). In IS, dysbiosis‐induced vagal dysfunction impairs the cholinergic anti‐inflammatory reflex, though experimental vagal nerve stimulation demonstrates therapeutic potential by reducing infarct volume and stabilizing intestinal barrier integrity (Deng et al. 2024). The enteric nervous system (ENS), comprising approximately 500 million neurons, constitutes a sophisticated “second brain” that maintains intestinal homeostasis through intimate microbiota interactions. Post‐stroke dysbiosis disrupts ENS function, precipitating increased intestinal permeability and bacterial translocation, which subsequently amplifies neuroinflammation via microglial activation (Pluta et al. 2021). Emerging evidence highlights a microbiota‐neuroepithelial axis, wherein chemosensory epithelial cells interface with sensory neurons to regulate gastrointestinal (GI) and CNS homeostasis (Ohara and Hsiao 2025). Additionally, the trigeminal pathway provides a direct anatomical route for periodontal pathogens, particularly *Porphyromonas gingivalis*, to access cerebral structures (Tao et al. 2024). Bacterial LPSs and extracellular vesicles have been identified in trigeminal ganglia and hippocampus following experimental gingival exposure, with neurectomy preventing such translocation (X. Ma et al. 2023). This pathway assumes particular clinical significance given the elevated prevalence of periodontal disease among IS patients and its association with stroke recurrence and cognitive deterioration (Lu et al. 2022).

### 3.3 Immune System

The immune system plays a pivotal role in gut–brain axis signaling, with gut microbiota critically modulating both central nervous system immune cell maturation and peripheral immune activation (Donald and Finlay 2023). Following IS, compromised gut barrier integrity triggers a cascade of immunological responses with distinct temporal and cellular characteristics. Intestinal mast cells undergo rapid activation, releasing histamine and pro‐inflammatory mediators that amplify systemic and central inflammation (Conesa et al. 2023), with age‐dependent mechanisms potentially contributing to poor outcomes in elderly patients (Blasco et al. 2020). Stroke‐induced alterations in T cell homeostasis, particularly shifts in Th1/Th2 balance and Th17 differentiation (Jian et al. 2019; D. Zhang et al. 2021; Lukasik et al. 2023), are substantially influenced by microbiota‐derived metabolites, especially SCFAs (Calvo‐Barreiro et al. 2023). Furthermore, brain‐resident microglia respond to both local injury signals and systemic inflammatory cues, with their activation states modulated by gut microbiota through metabolite signaling and vagal pathways (Erny et al. 2015; Fung et al. 2017; Eldahshan et al. 2019). Emerging evidence demonstrates that post‐stroke gut dysbiosis exacerbates microglial pro‐inflammatory polarization, thereby intensifying neuroinflammation (Y. Wang, Leak, et al. 2022). This bidirectional neuroimmune communication underscores the therapeutic potential of targeting gut microbiota to modulate post‐stroke immune responses and improve clinical outcomes.

### 3.4 Blood–Brain Barrier (BBB) Integrity

The BBB represents a critical interface in stroke pathophysiology, with its integrity intimately regulated by gut microbiota. Following IS, BBB disruption initiates a detrimental cascade wherein compromised gut permeability facilitates bacterial translocation and systemic release of pathogen‐associated molecular patterns (PAMPs) (S. Zhang, Chen, et al. 2024). LPS‐mediated activation of toll‐like receptor 4 (TLR4) signaling in cerebral endothelial cells directly compromises BBB integrity and amplifies neuroinflammation (S. Zhang, Chen, et al. 2024). Concurrently, post‐stroke gut dysbiosis elevates systemic inflammatory markers, including C‐reactive protein (CRP), LPS‐binding protein (LBP), and pro‐inflammatory cytokines, further perpetuating BBB dysfunction (Zheng et al. 2022). Emerging evidence underscores the regulatory role of microbiota‐derived metabolites, including SCFAs (Braniste et al. 2014), TMAO (Hoyles et al. 2021), tryptophan metabolites (Y. Huang et al. 2023), and ursodeoxycholic acid (UDCA) (Palmela et al. 2015)—in modulating BBB permeability. This MGBA operates bidirectionally (Xu et al. 2021), as acute stroke induces compositional shifts characterized by depletion of beneficial taxa (Lactobacillus and Bifidobacterium) and enrichment of pathogenic Enterobacteriaceae (Yamashiro et al. 2021). These alterations, coupled with stroke‐induced changes in gut motility and intestinal barrier function (J. Zhang, Ling, et al. 2024), establish a self‐perpetuating cycle of dysbiosis, systemic inflammation, and progressive neurological injury, highlighting microbiota‐targeted interventions as promising therapeutic strategies.

## 4 Pre‐Stroke Gut Microbiota Dysbiosis as an Upstream Risk Factor for IS

Growing evidence suggests that gut microbial imbalance may precede, rather than merely follow, IS, repositioning dysbiosis as an independent etiological contributor within the gut–brain–vascular axis (Hendel et al. 2026). At least three mechanistic pathways link pre‐stroke dysbiosis to cerebrovascular vulnerability.

The first involves microbial metabolite‐driven atherothrombosis. TMAO, produced via gut microbial *cutC/cutD*‐encoded trimethylamine (TMA) lyases and hepatic FMO3, promotes foam cell formation, impairs reverse cholesterol transport, and enhances platelet reactivity, thereby accelerating atherothrombotic cascades relevant to large‐artery and cardioembolic (CE) stroke subtypes (Dolkar et al. 2024; Yin et al. 2015; M. Yu et al. 2025). Evidence comes from Zhu et al. through fecal microbiota transplantation (FMT) experiments in germ‐free mice, demonstrating that the TMAO pathway is sufficient to increase infarct volume and worsen functional outcomes (Zhu et al. 2021). Clinically, elevated TMAO predicts recurrent cardiovascular events post‐stroke and associates with first stroke risk in hypertensive patients (Haghikia et al. 2018) (Nie et al. 2018). Pre‐procedural TMAO independently predicts silent cerebral embolic infarcts after carotid artery stenting (Wu et al. 2018). A 2026 dose–response meta‐analysis identified a plasma TMAO threshold of 3.0 µmol/L as a potential risk cutoff for incident stroke (J. Zhang et al. 2026). Additionally, a 2025 prospective cohort study demonstrated that TMAO levels were highest in the CE stroke subtype with concomitant culprit artery stenosis, suggesting its potential utility as an etiological discriminator among stroke subtypes (Y. Wang et al. 2025). However, a 2025 umbrella review cautions that the epidemiological evidence remains low confidence, mainly due to residual confounding (especially renal function) and scarce prospective data from healthy cohorts (Obeid et al. 2025). Thus, whether lowering TMAO yields clinical benefit awaits adequately controlled trials. In parallel, PAGln potentiates platelet activation via adrenergic receptor signaling (Nemet et al. 2020); in AIS complicated by T2D, elevated PAGln has been shown to exacerbate infarction through neutrophil extracellular trap (NET) formation, a process transmissible by FMT (M. Wei et al. 2025).

Second, one pathway concerns gut barrier disruption and systemic endotoxemia. Depletion of SCFA‐producing taxa—including Roseburia intestinalis, Faecalibacterium prausnitzii, and Lachnospiraceae—weakens tight junction integrity and permits LPS translocation into the systemic circulation (Koszewicz et al. 2021; Peh et al. 2022). Chronic TLR4 activation subsequently drives endothelial dysfunction, blood–brain barrier disruption, and microglial priming (S. Zhang, Chen, et al. 2024). In AIS patients, circulating LPS correlates with National Institutes of Health Stroke Scale (NIHSS) score (*r* = 0.31; *p* = 0.002) and independently predicts 3‐month poor outcomes and stroke mortality (Blaz et al. 2024). Notably, intestinal epithelial‐specific TLR4 knockout conferred greater neuroprotection than brain‐specific knockout in experimental models, implicating the gut as a primary pathological locus (X. Huang et al. 2026). Restoration of gut barrier integrity through microbiota‐targeted interventions, particularly dietary fiber‐derived polysaccharides, has been shown to reduce endotoxemia and attenuate neuroinflammation following IS (Xu et al. 2021).

The third pathway reflects comorbidity‐associated microbial pre‐conditioning. Hypertension, T2DM, obstructive sleep apnea, and metabolic syndrome each impose characteristic dysbiotic signatures characterized by SCFA‐producer depletion, pathobiont enrichment, and heightened systemic inflammatory tone (Jama et al. 2023; Mostafavi Abdolmaleky and Zhou 2024; X. Zhou et al. 2025). T2DM‐AIS patients exhibit higher NIHSS scores alongside greater butyrate‐producer depletion and elevated circulating LPS (H. Wang et al. 2021). Aging further amplifies this vulnerability: a roughly ninefold increase in the Firmicutes‐to‐Bacteroidetes ratio in aged mice was associated with worse post‐stroke mortality, an effect reversed by young‐donor FMT (Spychala et al. 2018).

Taken together, these pathways are mutually reinforcing and collectively lower the ischemic threshold before stroke onset. This pre‐stroke microbial priming phase provides the conceptual basis for understanding acute‐phase microbial–neural crosstalk and recovery‐phase microbial reprogramming, which are addressed in subsequent sections.

## 5 Gut Microbiota in the Acute Phase of IS

The gut microbiota has been demonstrated to be intimately associated with the onset and progression of stroke, yet IS can also rapidly induce gut microbiota dysbiosis, which in turn exacerbates cerebral infarction.

### 5.1 The Change of Gut Microbiota

IS induces rapid and profound alterations in gut microbiota composition, though clinical findings exhibit heterogeneity attributable to confounding variables including age, diet, medications, and genetic factors. While α‐diversity assessments yield inconsistent results across human cohorts, β‐diversity shifts and taxonomic compositional changes remain remarkably consistent (Yin et al. 2015; W. Wang et al. 2018; H. Li et al. 2020; Ling et al. 2020; Xiang et al. 2020; Guo et al. 2021; Haak et al. 2021). Meta‐analyses identify 62 upregulated and 29 downregulated microbial taxa in AIS patients, characterized predominantly by Proteobacteria enrichment and depletion of beneficial genera including Bacteroides, Prevotella, and Faecalibacterium (Yin et al. 2015; H. Li et al. 2020; Ling et al. 2020; Haak et al. 2021). Conversely, Firmicutes, particularly SCFA‐producing taxa such as Roseburia and Lachnospiraceae, demonstrate significant reductions (W. Wang et al. 2018; Xiang et al. 2020; Guo et al. 2021; Peh et al. 2022). Animal models provide temporally controlled evidence, revealing detectable dysbiosis within 24 h post‐stroke that persists for at least 7 days (Chelluboina et al. 2022; Kerr et al. 2022; Silva de Carvalho et al. 2022). Middle cerebral artery occlusion (MCAO) studies consistently demonstrate decreased α‐diversity, elevated Proteobacteria and Actinobacteria, and diminished Firmicutes (Spychala et al. 2018; Guan et al. 2023; F. Zhang, Deng, et al. 2024), alongside expansion of pathogenic Enterobacteriaceae (Tan et al. 2021). Emerging data indicate concomitant gut virome alterations (Chelluboina et al. 2022). Mechanistically, reduced SCFA production compromises gut and BBB integrity and microglial homeostasis (Braniste et al. 2014), while elevated LPS activates TLR4‐mediated neuroinflammation (S. Zhang, Chen, et al. 2024). Additionally, perturbed tryptophan metabolism favoring kynurenine pathways and attenuated vagal anti‐inflammatory signaling further exacerbate stroke pathophysiology (Y. Huang et al. 2023).

Human stroke microbiome findings show substantial inter‐study heterogeneity, often reflecting uncontrolled confounders rather than stroke effects alone. Key sources of variability include (i) diet, a dominant determinant of baseline microbiota (e.g., fiber, animal protein, and fermented foods) that is inconsistently captured across cohorts; (ii) antibiotics, frequently used for stroke‐associated pneumonia (SAP), which can rapidly and profoundly reshape microbial communities and may mask or mimic stroke‐related dysbiosis; (iii) proton‐pump inhibitors (PPIs), commonly prescribed during hospitalization, which alter gastric acidity and are associated with downstream shifts in gut taxa (including enrichment of oral‐origin and acid‐tolerant organisms); (iv) feeding route, as dysphagia‐driven enteral tube feeding differs from oral feeding in microbial seeding and nutrient delivery, producing route‐specific signatures; and (v) stroke severity (e.g., NIHSS/infarct burden), which modifies dysbiosis via autonomic dysfunction, immobility, and stroke‐induced immunodepression. These factors likely explain a meaningful proportion of between‐study inconsistencies and should be prospectively documented and incorporated as stratification variables or covariates (dietary assessment, antibiotic/PPI exposure, feeding route, and severity matching) in future human studies.

#### 5.1.1 Gut Microbiota and Subtypes of AIS

Emerging evidence reveals that gut microbiota alterations in AIS exhibit remarkable heterogeneity across distinct etiological subtypes, with compositional differences potentially reflecting subtype‐specific pathophysiological mechanisms and offering novel diagnostic and therapeutic opportunities. While stroke patients generally demonstrate reduced α‐diversity and distinctive β‐diversity patterns compared to healthy controls (Xiang et al. 2020), conflicting evidence exists, as lacunar cerebral infarction (LCI) patients showed preserved α‐diversity despite profound β‐diversity alterations in one study (J. Ma et al. 2024), suggesting compositional shifts may be more sensitive indicators than diversity metrics alone.

Lacunar infarction patients present distinctive microbial signatures characterized by the highest relative abundance of Fusobacteria and Cyanobacteria among stroke subtypes, with significant enrichment of Bacteroidaceae and Erysipelotrichaceae families (Guo et al. 2021). These patients demonstrate a consistent enterotype transition from Bacteroides‐dominated (enterotype 3) to Escherichia‐Shigella‐enriched (enterotype 1) communities, alongside elevated pro‐inflammatory taxa including Lactobacillus, Streptococcus, and Veillonella, concurrent with depletion of beneficial genera such as Agathobacter and Lachnospiraceae_UCG‐004 (X. Ma et al. 2023).

Large artery atherosclerotic (LAA) stroke patients exhibit higher gut microbial diversity with fundamentally altered community structures characterized by decreased Bacteroidetes and increased Firmicutes, Proteobacteria, and Actinobacteria (Xu et al. 2021). These patients demonstrate expansion of 41 genera, particularly Parabacteroides, Klebsiella, and Bifidobacterium, alongside an enterotype shift from Prevotella‐type to Ruminococcus‐type communities. Importantly, LAA patients exhibit significantly elevated plasma TMAO levels, implicating microbiota‐dependent metabolites in atherothrombotic pathogenesis.In contrast, CE stroke presents a unique profile wherein no significant gut microbiota compositional differences are detected between patients and controls (Xu et al. 2021), suggesting cardiac‐origin emboli may operate through microbiota‐independent mechanisms. However, CE patients share elevated TMAO levels with LAA patients despite distinct microbiota profiles, indicating potential common metabolic pathways independent of specific microbial compositions. Cryptogenic stroke (CS) patients demonstrate paradoxically increased α‐diversity with family‐level enrichment of Enterobacteriaceae, Streptococcaceae, and Lactobacillaceae, along with genus‐level expansion of Escherichia‐Shigella, Streptococcus, Lactobacillus, and Klebsiella (Zheng et al. 2022).

Recent thrombus microbiome characterization through metagenomic next‐generation sequencing reveals additional subtype‐specific signatures (X. Chen, He, et al. 2024). While α‐diversity metrics remain comparable between LAA and CE thrombi, LAA specimens demonstrate significant enrichment of *Ralstonia insidiosa* and 77 additional genera including Bradyrhizobium, Novosphingobium, and Cupriavidus, suggesting microbiota may directly populate atherosclerotic plaques and contribute to thrombus composition. These subtype‐specific microbial signatures underscore the importance of precision medicine approaches in microbiota‐targeted stroke prevention and treatment strategies.

#### 5.1.2 Gut Microbiota and AIS and Related Comorbidities

The mechanistic significance of comorbidity‐associated gut dysbiosis extends far beyond epidemiological co‐occurrence. Established stroke risk factors—hypertension, type 2 diabetes mellitus (T2DM), obstructive sleep apnea hypopnea syndrome (OSAHS), and non‐alcoholic fatty liver disease (NAFLD) (W. T. Zhang et al. 2020; H. Wang et al. 2021; J. Chen et al. 2022; Y. Jiang, Liu, et al. 2023; G. Yu et al. 2023)—each impose condition‐specific pre‐stroke dysbiotic signatures. Whether these signatures collectively constitute a “microbial priming state” that worsens ischemic injury remains an open question; nonetheless, two mechanistic convergence points recur across conditions: depletion of SCFA‐producing taxa driving gut barrier failure and subsequent LPS–TLR4–NF‐κB (nuclear factor kappa B) neuroinflammatory activation (J. Lee et al. 2020; Tan et al. 2021), and elevation of pro‐atherogenic metabolites such as TMAO and PAGln promoting atherothrombosis (Yin et al. 2015; Dolkar et al. 2024; M. Yu et al. 2025). This mechanistic framing positions comorbidity‐associated dysbiosis not as a peripheral association but as a potentially contributory component of the gut–brain–vascular axis in stroke pathogenesis. However, causal directionality and confounding by medications and diet remain major challenges in human studies (Table 1).

**TABLE 1:** Comorbidity‐associated microbial patterns and mechanisms.

Comorbidity	Typical microbial shifts (acute IS)	Putative mechanisms	Clinical signal
T2D	↓SCFA producers (Lachnospira, Blautia, Butyricicoccus), ↑LPS	Barrier injury, endotoxemia, Th17 skew	Higher NIHSS, worse outcomes
NAFLD	↓Richness; ↓Dorea/Dialister; ↑Enorma	Gut–liver–brain inflammation, bile acid dysregulation	Poorer function, metabolic complications
OSAHS	↑Bifidobacterium/Anaerostipes (varies)	Intermittent hypoxia niche, systemic inflammation	Higher infection/arrhythmia risk
Hypertension/HTPR	Altered Bacteroides/Ruminococcus; ↓diversity in HTPR	Microbiome–drug interaction, platelet reactivity	Antiplatelet resistance

Abbreviations: HTPR, high on‐treatment platelet reactivity; NAFLD, non‐alcoholic fatty liver disease; OSAHS, obstructive sleep apnea hypopnea syndrome; T2D, type 2 diabetes.

Contemporary evidence has established that AIS patients with concurrent comorbidities exhibit distinct gut microbiota alterations that extend beyond simple dysbiosis, representing condition‐specific microbial signatures with profound implications for disease progression and therapeutic responsiveness (J. Chen et al. 2022; Zheng et al. 2022; G. Yu et al. 2023). These findings collectively support a paradigm shift wherein gut microbiota functions simultaneously as a biomarker for disease severity stratification and a therapeutic target for precision intervention (Xia et al. 2019; Xiang et al. 2020; H. Wang et al. 2021; Su et al. 2023).

1. *Cardiovascular Comorbidities and Microbiota Modulation*

High on‐treatment platelet reactivity (HTPR) represents a critical challenge in secondary stroke prevention; emerging evidence suggests a potential link between gut microbiota composition and the efficacy of antiplatelet therapy. Patients exhibiting HTPR demonstrate significantly diminished microbial diversity accompanied by reduced relative abundance of unidentified Clostridia and Ralstonia compared to non‐HTPR counterparts (X. Chen, He, et al. 2024). These compositional shifts suggest that specific bacterial taxa may influence clopidogrel bioactivation, potentially via modulation of intestinal or hepatic CYP enzymes. Although these findings are preliminary and require functional validation, they highlight the promise of microbiota‐targeted strategies as a novel approach to overcome antiplatelet resistance in stroke patients.

Hypertension‐associated dysbiosis—characterized by depletion of Faecalibacterium and Lachnospiraceae and enrichment of Enterococcaceae (J. Chen et al. 2022; Y. Jiang, Liu, et al. 2023)—is hypothesized to mechanistically converge on SCFA deficiency and gut barrier compromise, creating a systemic endotoxemia‐primed vascular phenotype that amplifies both hypertension‐mediated vascular injury and post‐ischemic neuroinflammation. This gut‐vascular inflammatory axis, rather than descriptive microbial compositional differences per se, constitutes a potential mechanistic link through which hypertension‐associated dysbiosis increases stroke vulnerability.

1. *Sleep Disorders: Complex Microbiota–Neurometabolic Interactions*

The mechanistic significance of OSAHS‐associated dysbiosis in stroke pathogenesis lies in the capacity of chronic intermittent hypoxia (CIH) to create a stroke‐specific microbial vulnerability. This process is thought to be mediated, at least in part, by HIF‐1α–driven pathways, which selectively deplete obligate anaerobic SCFA producers (including members of Lachnospiraceae and Ruminococcaceae) that are exquisitely oxygen‐sensitive, while simultaneously promoting the expansion of facultative anaerobic pathobionts. This hypoxia‐driven remodeling of the microbial ecosystem impairs intestinal epithelial tight junction protein expression (ZO‐1, occludin), promotes LPS translocation, and primes the TLR4‐NF‐κB neuroinflammatory cascade—a well‐established mechanism that amplifies ischemic brain injury in the acute stroke phase (W. T. Zhang et al. 2020). In this context, OSAHS‐associated dysbiosis is not merely a co‐occurring microbiome alteration but a mechanistically coherent upstream contributor to the endotoxemia‐neuroinflammation axis central to this review.

1. *Metabolic Comorbidities: The Gut–Liver–Brain Axis*

Despite their distinct etiologies, T2DM, NAFLD, and hyperuricemia share a broadly similar mechanistic trajectory relevant to stroke: butyrate‐producer depletion undermines gut barrier integrity, enabling systemic endotoxemia to amplify post‐ischemic neuroinflammation. This convergence is conceptually compelling, though the extent to which each condition contributes independently—versus via shared metabolic dysfunction—has not been systematically disentangled.

NAFLD‐associated dysbiosis in AIS patients (reduced microbial richness and depletion of Dorea and Dialister) mechanistically disrupts the gut–liver–brain axis: hepatic steatosis‐driven bile acid metabolism alterations reduce secondary bile acid‐mediated FXR/Takeda G protein‐coupled receptor 5 (TGR5) signaling that normally maintains intestinal barrier integrity, thereby amplifying LPS translocation and systemic neuroinflammation in the context of IS (G. Yu et al. 2023). While the role of bile acid‐FXR/TGR5 signaling in NAFLD‐associated stroke is mechanistically plausible and supported by preliminary evidence (G. Yu et al. 2023), functional validation through targeted interventions (e.g., FXR agonists or microbiota‐mediated bile acid modulation) in stroke models remains an important direction for future research.

Hyperuricemia‐associated dysbiosis converges on the same mechanistic axis through selective depletion of Parabacteroides distasonis—a keystone SCFA producer with established anti‐inflammatory and gut barrier‐protective properties. Critically, *P. distasonis* depletion in hyperuricemic stroke models results in exacerbated neuroinflammation, impaired BBB integrity, and increased oxidative stress, while targeted *P. distasonis* supplementation reverses these pathological changes and improves neurological outcomes (H. Wei et al. 2024)—providing mechanistic validation that the purine metabolism–microbiota interaction operates through the same gut–barrier–neuroinflammation axis central to this review.

T2D patients with AIS present more severe stroke manifestations (higher NIHSS scores) accompanied by notably decreased butyrate‐producing genera, including Lachnospira, Blautia, and Butyricicoccus, alongside reduced fecal butyrate concentrations and elevated serum LPS and D‐lactate levels, collectively indicating compromised gut barrier integrity (H. Wang et al. 2021). Beyond this butyrate–depletion–endotoxemia axis, emerging evidence identifies a second mechanistically distinct pathway in T2D–AIS: elevated circulating PAGln, driven by T2D‐associated gut compositional shifts (enrichment of Enterobacteriaceae, Verrucomicrobiota, and Klebsiella), exacerbates cerebral infarction through NET formation, systemic inflammation, and oxidative stress—a causal relationship demonstrated through FMT from T2D–AIS patients to rodent stroke models (M. Wei et al. 2025). This dual‐axis mechanism (butyrate‐depletion–endotoxemia + PAGln–NET) positions T2DM as the comorbidity with the most mechanistically elaborated gut‐mediated amplification of IS severity.

Animal studies provide crucial mechanistic validation, demonstrating that pre‐existing metabolic conditions create a “primed” microbiota environment that undergoes more severe dysbiosis following cerebral ischemia. Diabetic mouse models consistently demonstrate stroke‐induced profound loss of butyrate‐producing bacteria (Lachnospiraceae and Ruminococcaceae), resulting in decreased SCFA levels, impaired intestinal barrier function, and enhanced inflammation. Sodium butyrate administration partially reverses these pathological changes, and Huang‐Lian‐Jie‐Du Decoction (HLJDD) achieves similar restoration through TLR4/NF‐κB pathway inhibition (H. Wang et al. 2021; C. Chen et al. 2025)—validating the therapeutic tractability of comorbidity‐primed dysbiosis as a target for precision stroke intervention.

Collectively, these comorbidity‐specific findings converge on four shared pathological features—(i) SCFA‐producer depletion, (ii) pro‐inflammatory pathobiont enrichment, (iii) compromised gut barrier integrity, and (iv) systemic inflammatory sensitization—that mechanistically underpin the “pre‐stroke microbial vulnerability state” and the acute‐phase neuroinflammatory amplification described throughout this review. Rather than representing tangential associations, comorbidity‐associated dysbiosis constitutes a mechanistically coherent upstream layer of the gut‐brain‐vascular axis, providing the biological rationale for comorbidity‐stratified microbiome profiling and precision intervention in IS.

#### 5.1.3 Gut Microbiota and Host Factor

Host determinants—including age, sex, and genetic/epigenetic architecture—critically modulate gut microbiota composition and function, thereby shaping IS susceptibility, severity, and recovery trajectories through integrated regulation of intestinal permeability, immune homeostasis, and metabolite signaling pathways.

1. *Age*

Age‐related microbiota remodeling represents a pivotal nexus linking physiological senescence to cerebrovascular risk. Aging is characterized by progressive erosion of microbial diversity, expansion of pro‐inflammatory taxa (Proteobacteria and Enterobacteriaceae), and depletion of beneficial genera (Bifidobacterium and Lactobacillus), collectively fostering “inflammaging” and metabolic dysfunction (O'Toole and Jeffery 2018). Stroke superimposes acute dysbiosis upon this aged microbiome, establishing a pathological feedback loop. Meta‐analytic evidence demonstrates that elderly stroke patients exhibit more profound α‐diversity reduction and pathobiont enrichment compared to younger cohorts and age‐matched controls (Y. Lee et al. 2021; Peh et al. 2022). Critically, age‐dependent alterations in microbial metabolites—SCFAs and bile acids—correlate with neurological deficit severity and predict GI complications and nosocomial infections (Roth et al. 2020; Henry et al. 2021), risks further amplified by comorbidities such as diabetes and chronic kidney disease (M. Wang, Wang, et al. 2022). Mechanistic validation derives from aged murine models (18–20 months), wherein stroke induces catastrophic loss of SCFA‐producing taxa alongside pro‐inflammatory bacterial expansion (J. Lee et al. 2020). Remarkably, FMT from young donors restores intestinal barrier integrity, attenuates neuroinflammation, and enhances functional recovery (J. Lee et al. 2020; J. Lee et al. 2023). Targeted SCFA supplementation or bacterial consortia administration similarly mitigates microglia‐driven immune activation, while estradiol supplementation in aged females modulates microbial architecture and ameliorates barrier disruption (J. Lee et al. 2023; H. Wei et al. 2024), underscoring age‐tailored microbiome interventions as promising therapeutic avenues.

2. *Sex*

Sex‐specific microbiota‐brain interactions emerge through endocrine–immune–microbial crosstalk. Male rodents demonstrate earlier and more severe post‐stroke gut barrier failure with increased mortality and worse sensorimotor outcomes, whereas females exhibit relative protection (Ahnstedt et al. 2020; El‐Hakim et al. 2021). Causality is established through FMT experiments: female‐derived microbiota confers smaller infarcts, improved survival, elevated SCFAs and tryptophan metabolites, and reduced pro‐inflammatory cytokines, associated with lower Firmicutes‐to‐Bacteroidetes ratios (J. Wang, Zhong, et al. 2022). Sex‐divergent epithelial defenses manifest as male‐predominant antimicrobial peptide upregulation (Reg3 family) versus female‐enhanced mucin fortification (Muc2/Muc4), with estradiol in aged females restoring mucin programs and enriching lactobacilli/bifidobacteria (J. Lee et al. 2023). Reproductive senescence reshapes female microbiomes, and young estrogen‐treated donor microbiota reduces infarcts in middle‐aged recipients (Park et al. 2020). Xenometabolites exhibit sex dimorphism—TMAO demonstrates differential platelet activation and thrombotic potential (Lucas et al. 2023), while androgens modulate bile acid–microbiota circuits influencing stroke outcomes (Spiegel 2024). This evidence supports a SEX framework (systemic inflammation regulation, epithelial barrier maintenance, and xenometabolite modulation) governing sex‐specific microbiota–brain axis function in stroke.

3. *Genetic and Epigenetic Factors*

Genetic and epigenetic determinants further sculpt microbial ecosystems and stroke phenotypes. Fucosyltransferase 2 (FUT2) non‐secretor status reduces mucosal fucosylation, depletes Bifidobacterium and butyrate producers (Yatsunenko et al. 2012; Lopera‐Maya et al. 2022), and associates with pro‐inflammatory profiles elevating IS risk (F. Xu et al. 2020). Immune‐sensing polymorphisms (TLR4 and NOD2) tune microbial recognition and intestinal permeability, modulating neuroinflammation (Erny et al. 2015; M. Wang, Pan, et al. 2022), while bile acid pathway genes (CYP7A1, FXR, and TGR5) govern primary–secondary bile acid transformations affecting blood–brain barrier integrity (Fiorucci and Distrutti 2015; Yanguas‐Casás et al. 2017; Chiang and Ferrell 2019). Pharmacogenomic–microbiome interactions influence clopidogrel activation and statin metabolism (Weersma et al. 2020; Pant et al. 2023). Microbial metabolites function as epigenetic mediators: butyrate inhibits histone deacetylases (HDACs) to promote microglial maturation and regulatory T‐cell induction (Furusawa et al. 2013; Masuda et al. 2020); tryptophan derivatives and bile acids modulate sirtuins/PARP and chromatin architecture (Spichak et al. 2021; Y. Huang et al. 2023).

In the future, the concept of “precision symbiosis” should guide therapeutic development: genotype‐informed prebiotic strategies (e.g., FUT2‐aware bifidogenic approaches), SCFA augmentation, bile acid receptor modulators, and epigenetic clocks incorporating microbial metabolite signatures for risk stratification and treatment timing. Single‐cell multi‐omics of gut‐brain immune circuits will further refine this microbiome–epigenome–immune therapeutic axis.

### 5.2 Gut Microbiota and Complications of Poststroke

Post‐stroke complications—particularly SAP and GI dysfunction—constitute major determinants of morbidity and mortality, with accumulating evidence establishing gut microbiota dysbiosis as a central, modifiable pathophysiological mechanism.

SAP, affecting up to 30% of IS patients, extends beyond aspiration‐related pathways. Metagenomic investigations demonstrate that a substantial fraction of airway bacteria originates from intestinal commensals rather than oropharyngeal flora (Kalra et al. 2015), challenging traditional paradigms. Prospective cohort studies reveal that early depletion of butyrate‐producing taxa independently predicts post‐stroke infection risk (Haak et al. 2021); Chinese pilot cohorts establish SAP as characterized by Roseburia depletion and pathobiont enrichment, with gut dysbiosis independently associating with 30‐day mortality and 90‐day poor outcomes, achieving biomarker performance comparable to clinical risk scores (Xia et al. 2021; Z. Li et al. 2023; J. Chen, Gao, et al. 2024). The oral–gut–lung axis further extends this framework: smaller cohorts link oral pathobionts (Neisseria, Porphyromonas, and Prevotella) to SAP susceptibility (Cieplik et al. 2020), with recent reviews underscoring the clinical relevance of oral microbiome assessment (Azzolino et al. 2025). SARS‐CoV‐2 co‐infection in stroke patients aggravates dysbiosis (elevated Enterobacteriaceae, diminished Ruminococcaceae/Lachnospiraceae) and worsens recovery (N. Chen et al. 2020), suggesting synergistic virus–host–microbiome interactions.

Mechanistic elucidation demonstrates that post‐stroke pulmonary infections arise from intestinal commensals, particularly Enterobacteriaceae, mediated by barrier failure and impaired motility (Stanley et al. 2016). Stroke disrupts neuro‐immuno‐metabolic homeostasis, driving complement activation, NF‐κB signaling, Treg depletion, and polarization toward pathogenic γδT/Th1/Th17 phenotypes, alongside intestinal SCFA and bile acid depletion (Díaz‐Marugan et al. 2023). Critically, selective NF‐κB inhibition curtails gut Enterobacteriaceae expansion but fails to prevent pulmonary colonization, indicating that integrated targeting of barrier integrity, immune modulation, and metabolite restoration is essential (Díaz‐Marugan et al. 2023).

Therapeutic paradigms are evolving toward precision microbiome engineering: probiotics/synbiotics and fermentable fibers to enrich butyrate producers; FMT demonstrating ecological restoration in preclinical and pilot studies (Hediyal et al. 2024); SCFA‐axis strategies—postbiotic butyrate/propionate or FFAR2/FFAR3/GPR109A agonism; integrated oral–gut–lung care coupling oral hygiene with targeted prebiotics (Clottes et al. 2023); mucosal immune modulation combining gut‐selective NF‐κB inhibitors with Treg‐enhancing postbiotics (Amin et al. 2023; Díaz‐Marugan et al. 2023). Future priorities include multi‐omic‐stratified RCTs and rapid (24–48 h) ecological–metabolomic profiling to enable personalized intervention bundles.

Post‐stroke GI dysfunction affects approximately 50% of patients, manifesting as constipation, upper GI bleeding, and malnutrition, all correlating with adverse outcomes (Cheng et al. 2020; Camilleri 2021). Clinical profiling reveals dysbiosis—depleted SCFA producers (Faecalibacterium, Roseburia), expanded Enterobacteriaceae, reduced SCFAs, altered bile acids, and elevated TMAO—associated with barrier injury, dysmotility, infection, and poor functional recovery (Camilleri 2021; Peh et al. 2022). Stroke severity, advanced age, polypharmacy (opioids, anticholinergics, and PPIs), and antibiotics exacerbate these perturbations (Cheng et al. 2020; Camilleri 2021). Phenotype‐driven stratification into “motility‐dominant,” “mucosal‐injury‐dominant,” and “infection‐prone” subtypes based on microbe–metabolite signatures is emerging.

Experimental models delineate a bidirectional brain–gut–immune loop: cerebral ischemia induces autonomic dysregulation and inflammatory cascades that disrupt tight junctions within two hours, promoting bacterial translocation (Benakis et al. 2016; Singh et al. 2016; Díaz‐Marugan et al. 2023; H. Wang, Li, et al. 2024). Microbial metabolites signal via G protein‐coupled receptor 41/43 (GPR41/43), FXR/TGR5, and aryl hydrocarbon receptor (AhR) to modulate enteric neuromuscular function and mucosal immunity (Benakis et al. 2016; Díaz‐Marugan et al. 2023). Germ‐free and FMT studies confirm microbiota‐dependent T‐cell reprogramming and neuroinflammatory feedback (Benakis et al. 2016; Spychala et al. 2018).

Evidence‐based management integrates foundational care—early enteral nutrition, mobilization, dysphagia rehabilitation, standardized constipation protocols, and antibiotic stewardship (Cheng et al. 2020; Camilleri 2021)—with mechanism‐targeted interventions: SCFA supplementation, fermentable fiber, probiotics, bile‐acid‐receptor modulators (Benakis et al. 2016; Spychala et al. 2018; Díaz‐Marugan et al. 2023), neuromodulation (vagus‐nerve stimulation), and standardized FMT/dietary programs (Xia et al. 2019; Yamashiro et al. 2021). Stratified RCTs guided by microbiome–metabolite biomarkers are essential to validate phenotype‐specific regimens and advance precision post‐stroke complication management.

### 5.3 Limitations and Inconsistencies

Despite the mechanistic coherence of the findings reviewed above, five key methodological limitations must be acknowledged. First, the predominance of cross‐sectional designs precludes establishing temporality: whether pre‐existing dysbiosis drives stroke susceptibility, or acute ischemic injury itself induces dysbiosis, remains an unresolved question of reverse causation that prospective cohort studies and Mendelian randomization analyses have only partially addressed (Dang et al. 2021). Second, inter‐cohort taxonomic inconsistencies undermine generalizability; the Enterobacteriaceae findings are illustrative—while most studies report enrichment as a consistent dysbiosis feature, at least one meta‐analysis reported net reductions (Hu et al. 2023), likely reflecting geographic dietary variation, antibiotic exposure histories, and 16S rRNA methodological differences. No universal “stroke dysbiosis signature” has yet been established. Third, 16S rRNA sequencing—the dominant methodology—restricts identification to genus level, inadequately capturing strain‐specific metabolic functions (butyrate production, TMAO generation, and LPS biosynthesis); shotgun metagenomics and targeted metabolomics should be prioritized in future work. Fourth, the vast majority of mechanistic evidence derives from inbred rodent MCAO models that fail to recapitulate the etiological, genetic, and comorbidity heterogeneity of human stroke; sex imbalance in preclinical cohorts’ further limits generalizability given established sex differences in microbiota composition and ischemic immune responses. Fifth, confounding by co‐administered medications—antibiotics, proton pump inhibitors, statins, and antihypertensives—each with documented independent effects on microbiota composition, represents a pervasive and incompletely controlled bias in clinical studies (Nagano et al. 2025). Future investigations must prioritize prospective biobank cohorts with pre‐stroke microbiome profiling, multi‐omics integration, sex‐stratified designs, and medication‐adjusted analytical frameworks to establish causally interpretable microbiota–stroke relationships.

## 6 Gut Microbiota in the Chronic Phase of Stroke

The chronic phase of IS represents a critical window for functional recovery and rehabilitation, during which gut microbiota emerges as both a pivotal modulator of neurological outcomes and a promising therapeutic target. Stroke‐induced dysbiosis—characterized by profound shifts in microbial abundance, diversity, and metabolic function—persists well beyond the acute phase and significantly impacts recovery potential while contributing to prevalent complications, including post‐stroke cognitive impairment (PSCI) and post‐stroke depression (PSD).

### 6.1 Longitudinal Dynamics and Mechanistic Insights

Although longitudinal investigations remain limited, accumulating evidence demonstrates that gut dysbiosis persists chronically with gradual but incomplete restoration paralleling neurological recovery. Recent multicenter and prospective studies reveal that while partial normalization of microbial composition occurs within 6 months post‐stroke, significant deviations from healthy controls endure, notably persistent reductions in beneficial taxa such as Ruminococcaceae and Clostridiales alongside compensatory increases in SCFA‐producing genera, including Akkermansia and Bacteroides (Cai et al. 2023; Marsiglia et al. 2023). Critically, patients exhibiting faster microbial diversity recovery within 6 months generally achieve superior functional outcomes and reduced systemic inflammation (Peh et al. 2022). Conversely, sustained depletion of key SCFA producers—Faecalibacterium prausnitzii and Roseburia—correlates strongly with persistent elevation of inflammatory markers (IL‐6 and CRP) and poor prognosis (Xu et al. 2021).

Preclinical models establish causality: landmark investigations by J. Lee et al. (2020) demonstrated that stroke‐induced dysbiosis drives systemic and neuroinflammation, and that reintroducing specific SCFA‐producing bacteria during the chronic phase significantly reduces neuroinflammation, restores blood–brain barrier integrity, and enhances functional recovery. Moreover, MCAO studies in non‐human primates reveal persistent dysbiosis even 12 months post‐stroke compared to sham‐operated controls (Y. Chen et al. 2019), underscoring prolonged ecological disruption and its influence on chronic recovery. Collectively, these findings position early microbiota restoration as a pivotal strategy for modulating immune responses and promoting neurological rehabilitation.

### 6.2 PSCI

PSCI, a prevalent and debilitating complication affecting quality of life and limiting recovery, exhibits consistent microbiota signatures across cohorts: reduced α‐diversity (lower Simpson index) and compositional shifts, including enrichment of Proteobacteria and depletion of beneficial Firmicutes lineages (e.g., Clostridia, Lachnospiraceae) and key SCFA producers such as Faecalibacterium prausnitzii and Roseburia (Ling et al. 2020; H. Wang, Zhang, et al. 2022; Hu et al. 2023). Family/genus‐level patterns reveal higher Bacteroidaceae, Lachnospiraceae, and Veillonellaceae in PSCI, although findings for Enterobacteriaceae remain heterogeneous: a meta‐analysis reported reductions, whereas a Chinese cohort with incident PSCI showed elevations alongside lower fecal butyrate (H. Wang, Zhang, et al. 2022; Hu et al. 2023). Distinct signatures in PSCI with comorbid depression (PSCCID)—notably elevated Proteobacteria, Gammaproteobacteria, and Enterobacteriaceae—suggest subtype‐specific dysbiosis (Ling et al. 2020). Microbial patterns correlate with risk factors (baseline NIHSS, homocysteine, and brain atrophy) and cognitive performance (MMSE), with genera such as Prevotella, Blautia, and Ruminococcus linked to outcomes (Ling et al. 2020; Cui et al. 2023).

Mechanistic studies establish causality along convergent axes. In ischemia models, baicalin restored TMA/TMAO homeostasis, remodeled gut microbiota, suppressed neuroinflammation, and improved hippocampal plasticity and cognition, with antibiotic pretreatment abolishing benefits (J. Liu et al. 2020). Similarly, baicalein improved cognition in bilateral common carotid artery occlusion (BCCAO) vascular cognitive impairment models while modulating microbiota and dampening neuroinflammatory cytokines (Song et al. 2024). FMT from PSCI donors and SCFA supplementation experiments demonstrate that altered microbiota and SCFA depletion exacerbate cognitive impairment via neuroinflammation, synaptic dysfunction, and oxidative phosphorylation deficits, while FMT/SCFAs mitigate these phenotypes (Su et al. 2023). Mechanistically, PSCI mice exhibit Enterobacteriaceae expansion, intestinal TLR4 upregulation, elevated circulating LPS/LBP and pro‐inflammatory cytokines, reduced fecal butyrate, and intestinal damage; sodium butyrate reverses these alterations, and post‐stroke LPS exposure phenocopies PSCI pathology (H. Wang, Zhang, et al. 2022). Exercise preconditioning further improves cognition, attenuates inflammation, and normalizes microbial diversity/composition (Lv et al. 2022). A unifying model posits a Proteobacteria/Enterobacteriaceae–LPS–TLR4 axis that primes microglia and disrupts synaptic plasticity, counterbalanced by an SCFA‐butyrate axis restoring barrier function, epigenetic tone, and neuroglial homeostasis.

### 6.3 PSD

PSD, among the most common neuropsychiatric complications impairing quality of life and recovery, is consistently associated with reduced alpha diversity and compositional shifts, including relative Firmicutes depletion and Proteobacteria/Bacteroidetes enrichment; genus‐level signals include enrichment of Akkermansia and Streptococcus coupled with depletion of butyrate‐producing taxa such as Butyricicoccus and Holdemanella (Luo and Fang 2022; Yao et al. 2023). These patterns parallel functional SCFA deficits (notably butyrate and acetate), which support BBB integrity and neuroplasticity, with inadequacy linked to heightened neuroinflammation. Biomarkers of gut barrier injury (intestinal fatty acid binding protein [iFABP]) are elevated in PSD and, combined with lipid pathway disturbances (steroid biosynthesis), enable diagnostic models with strong discrimination (AUC 0.847) (Zhong et al. 2022). Evidence also points to tryptophan metabolic shifts favoring kynurenine production—a pro‐depressogenic route associated with microglial activation—bridging dysbiosis and neuroimmune activation.

Preclinical PSD models corroborate causality: rat paradigms reveal reduced microbial diversity with SCFA‐producing genera (e.g., Blautia) depletion, accompanied by fecal metabolomic disturbances in amino acid and lipid pathways (W. Jiang et al. 2021; W. Jiang, Chen, et al. 2023). Butyrate exerts antidepressant‐like effects via HDAC inhibition and BDNF upregulation (Suda and Matsuda 2022), with reduced fecal butyrate tracking hippocampal synaptic protein loss (PSD95 and synapsin‐1) and altered community composition (Gong et al. 2021). Microbiota‐centered interventions—electroacupuncture restoring Bifidobacterium/Lactobacillus and suppressing colonic NLRP3 with hippocampal cytokine reduction—ameliorate depressive behaviors (Cai et al. 2023). Environmental and nutritional modifiers further influence outcomes: pair‐housing normalizes diversity and attenuates inflammatory tone (reduced TNF‐α/IL‐1β/IL‐6 and elevated IL‐10), while inulin improves depressive‐like behaviors via gut remodeling and IGF‐1‐MAPK signaling; repetitive xenon therapy similarly improves affective behaviors alongside microbiota and cytokine remodeling (Dandekar et al. 2022; Shao et al. 2024).

### 6.4 Translational Implications and Therapeutic Strategies

Current evidence motivates a two‐step clinical pathway: risk stratification using composite microbiome–metabolite panels (Proteobacteria predominance, SCFA‐producer depletion, fecal butyrate, plasma TMAO/kynurenine/tryptophan ratio, LPS/LBP, and iFABP) to identify high‐risk phenotypes. It must be emphasized, however, that the evidence base supporting this pathway is predominantly derived from preclinical studies and observational data; prospective interventional human trials validating microbiota‐stratified treatment algorithms in stroke are lacking. Feasible near‐term modalities with at least preliminary clinical support include diet‐first fiber enrichment and judicious prebiotics (inulin), along with targeted probiotics/synbiotics, which have demonstrated modest safety and tolerability signals in stroke‐related trials. Postbiotics (sodium butyrate), structured aerobic exercise, and electroacupuncture have supportive preclinical and/or limited observational data, but require prospective controlled evaluation. FMT should currently be regarded as investigational, applicable only within the context of clinical trials given the absence of human efficacy data in stroke. Clinical trials should standardize intervention windows (subacute 2–12 weeks post‐stroke), employ domain‐specific cognitive and depression composites (MMSE and HAMD/PHQ‐9), longitudinally track mechanistic biomarkers (SCFAs, TMAO, kynurenine indices, LPS/LBP, iFABP, and cytokines), and adopt microbiota‐stratified adaptive designs enriching for responders (high Proteobacteria and low butyrate phenotypes). Critically, safety monitoring must address dysphagia, malnutrition, antibiotic exposure, and immunocompromise—prevalent comorbidities in post‐stroke populations. In particular, the stroke‐induced immunodepression syndrome (SIDS) that characterizes the acute phase creates a window of heightened vulnerability: live probiotic administration during this period carries an elevated risk of bacteremia in patients with compromised gut barrier integrity or central venous access, while FMT poses additional risks of donor‐pathogen transmission (including ESBL‐producing organisms and MDROs) in immunosuppressed recipients. These risks necessitate explicit safety stratification criteria in trial design: patients with severe immunosuppression (lymphocyte count <1.0 × 10^9^/L), active systemic infection, severe dysphagia (Functional Oral Intake Scale score ≤2), or recent (within 4 weeks) broad‐spectrum antibiotic exposure should be considered for exclusion or require enhanced safety monitoring protocols. Postbiotic (heat‐killed bacterial) preparations and washed microbiota transplantation (WMT) may represent safer alternatives to live probiotics and conventional FMT, respectively, in high‐risk stroke subpopulations, warranting dedicated evaluation in phase I safety trials.

### 6.5 Limitations in Post‐Stroke Complication Microbiota Research

Despite the mechanistic plausibility and convergent experimental support for gut dysbiosis in post‐stroke complications, four key limitations require acknowledgment. First, for PSCI, taxonomic heterogeneity remains unresolved: while most cohorts report Proteobacteria enrichment and SCFA‐producer depletion as consistent signatures, Enterobacteriaceae findings diverge critically—a prospective Chinese cohort demonstrated elevations alongside reduced fecal butyrate (H. Wang, Zhang, et al. 2022), whereas a meta‐analysis reported net reductions (Hu et al. 2023). This contradiction likely reflects the diagnostic heterogeneity of “PSCI” as a composite entity encompassing vascular cognitive impairment, post‐ischemic cognitive decline, and mixed dementia, each potentially associated with distinct microbial profiles. Subtype‐stratified analyses and domain‐specific cognitive instruments beyond MMSE/MoCA are necessary to resolve this inconsistency. Second, for PSD, the directionality of the microbiota‐depression relationship remains fundamentally ambiguous. The gut–brain axis is bidirectional: depression itself alters microbiota composition, creating circularity that cross‐sectional studies cannot resolve. While SCFA deficiency, serotonin pathway dysregulation, and kynurenine pathway activation provide mechanistically plausible intermediary mechanisms, prospective studies with longitudinal microbiota sampling bracketing depression onset are required to establish temporality. Third, for SAP, the mechanistic evidence linking gut dysbiosis to pulmonary immune suppression relies predominantly on indirect immunological endpoints—serum cytokines, T‐cell subsets, and complement activation—rather than direct demonstration of gut‐to‐lung bacterial translocation in human subjects. The confounding effects of nasogastric tube feeding, post‐stroke dysphagia, prophylactic antibiotics, and proton pump inhibitor use on gut microbiota composition in hospitalized stroke patients remain inadequately controlled in existing clinical studies. Fourth, across all three complications, a shared limitation is the reliance on single‐center observational studies with small sample sizes, short follow‐up durations, and inconsistent definitions of clinical endpoints (NIHSS, mRS, MMSE, and HAM‐D), limiting cross‐study comparability and meta‐analytic synthesis. Future investigations should adopt multicenter prospective designs, standardized microbiome profiling protocols, and validated composite clinical endpoints to enable causally interpretable conclusions.

## 7 Gut Microbiota and Therapeutic Applications in IS

The recognition that gut microbiota dysbiosis plays an important role in IS pathophysiology and recovery has spurred development of diverse microbiota‐targeted therapeutic strategies, ranging from conventional probiotics to innovative neuromodulation and traditional medicine approaches, which together offer a potential translational framework for stroke rehabilitation. It is important to note, however, that the overwhelming majority of evidence supporting these strategies derives from preclinical animal studies, predominantly rodent models of MCAO. Human clinical data—particularly from randomized controlled trials—remain scarce, and direct extrapolation from animal findings to clinical practice must be approached with caution. In the following sections, we explicitly distinguish preclinical from clinical evidence and highlight where gaps in human data necessitate further investigation.

These therapeutic strategies largely reflect the pathophysiological axes established in this review. Specifically, five mechanistic targets emerge from earlier sections and shape the therapeutic landscape: (i) the SCFA‐depletion→gut barrier failure→LPS‐TLR4‐NF‐kB neuroinflammatory axis, which may be targeted by probiotics, prebiotics, SCFA supplementation, and TCM formulas that restore butyrate‐producing taxa; (ii) the TMAO/PAGln‐mediated atherothrombotic axis, potentially addressable through dietary modulation and gut microbial community restructuring; (iii) the tryptophan‐kynurenine/serotonin neuroimmune axis, targeted by indole‐derivative supplementation and TCM tryptophan metabolism modulators; (iv) the bile acid‐FXR/TGR5 signaling axis, targeted by UDCA and bile acid receptor modulators; and (v) the vagal‐cholinergic anti‐inflammatory axis, targeted by vagus nerve stimulation (VNS) and neuromodulation. Table 2 summarizes the therapy‐to‐mechanism relationships across all therapeutic modalities reviewed in this section.

**TABLE 2:** Evidence grading (animal vs. human) for microbiota‐targeted interventions in ischemic stroke.

Intervention	Main rationale (MGBA target)	Animal evidence	Human evidence	Notes (translation/safety)
Dietary fiber/diet	SCFA restoration, barrier support	B	Pilot/Obs	Confounded by adherence; low risk
Probiotics/synbiotics	Taxa modulation, inflammation/SCFAs	B	Pilot/RCT (limited)	Caution in immunocompromised states
Prebiotics (inulin, resistant starch)	Substrate‐driven remodeling	B	None/limited	Safer than live probiotics; feeding route matters
FMT	Whole‐ecosystem restoration	A	None	Infection/MDRO risk; trial setting recommended
SCFA supplementation (butyrate)	Epigenetic and immune effects	B	None	Bioavailability and BBB penetration uncertain
Indole metabolites (e.g., IPA)	AhR/Treg, barrier protection	C	None	Early‐stage, preclinical
Bile‐acid signaling (e.g., UDCA)	TGR5/anti‐inflammasome	C	None (stroke‐specific)	Clinically used in liver disease, not validated in stroke
TCM compounds (e.g., ginsenoside Rg1, berberine)	Anti‐inflammatory + microbiome modulation	B–C	None (microbiome‐mediated)	Primarily mechanistic animal studies
Acupuncture/electroacupuncture	Neuroimmune+ microbiome modulation	B	RCTs for rehabilitation (microbiome endpoints lacking)	Microbiome linkage not established in humans
Neuromodulation (VNS, tDCS)	Vagal/metabolic pathways	B	RCTs/clinical use for rehabilitation (microbiome endpoints lacking)	Human microbiome mechanism unproven
Exercise	SCFA‐producing taxa + inflammation	B	Strong rehabilitation evidence; limited microbiome endpoints	High feasibility; confounded by severity/diet
Social/environmental factors (pair‐housing)	Stress–immune–microbiome axis	C	None	Preclinical evidence only

*Note*: Animal evidence: A = multiple independent studies/robust; B = several studies/moderate; C = limited/early. Human evidence: RCT = randomized trial; Pilot = small interventional study; Obs = observational association; None = no direct stroke data.

### 7.1 Probiotics, Prebiotics, and Synbiotics

Probiotic, prebiotic, and synbiotic interventions are thought to work partly by reversing the SCFA‐depletion→gut barrier failure→systemic endotoxemia cascade—one of the primary upstream pathophysiological axes considered in this review—by promoting butyrate‐producing taxa, enhancing intestinal tight junction protein expression, and suppressing LPS‐TLR4‐mediated neuroinflammatory signaling. Probiotic, prebiotic, and synbiotic interventions restore post‐stroke dysbiosis by promoting beneficial bacterial taxa, enhancing intestinal barrier integrity, and augmenting SCFA‐producing capacity. In preclinical studies, a landmark study in aged mice demonstrated that SCFA‐producing bacterial consortia (*Bifidobacterium longum*, *Clostridium symbiosum*, *Faecalibacterium prausnitzii*, and *Lactobacillus fermentum*) combined with prebiotic inulin significantly improved motor function, cognitive performance, and depression‐like behavior—effects comparable to young FMT (Holmes et al. 2020). These animal data support a possible link between microbial metabolite restoration and functional recovery through elevated SCFA levels and reduced neuroinflammation. In a separate line of work, Pueraria lobata root extract‐derived resistant starch (PLR‐RS) functioned as a prebiotic enriched Akkermansia and Bifidobacterium while promoting gut‐derived melatonin production, and was associated with amelioration of IS‐induced brain and intestinal damage (Lian et al. 2023). Critically, FMT from PLR‐RS‐treated rats replicated neuroprotective effects, suggesting the translational potential of prebiotic‐driven ecological remodeling. Translating these findings to humans, however, requires considerable caution: a small number of clinical trials have examined probiotic supplementation in stroke patients, with some reporting modest improvements in inflammatory markers and functional outcomes, but most are underpowered, short in duration, and lack standardized microbiome endpoints. Whether the specific bacterial consortia and metabolite targets identified in rodent models are relevant and achievable in human stroke patients remains to be demonstrated in adequately powered RCTs.

The prophylactic use of probiotics via the gut–spleen–brain axis further extends this mechanistic rationale: Lactobacillus strains (*L. reuteri* GMNL‐89 and *L. paracasei* GMNL‐133) preserved intestinal tight junction integrity, reduced BBB permeability, regulated splenic cytotoxic T cells and Th1/Th2 balance, and increased SCFA concentrations following permanent MCAO effects consistent with attenuation of gut barrier failure and peripheral immune dysregulation (H. Wang, Li, et al. 2024). This gut–spleen–brain mechanistic axis, wherein probiotic‐mediated gut barrier reinforcement appears to attenuate splenic immune activation and downstream neuroinflammation, offers a potential mechanistic rationale for prophylactic probiotic deployment in high‐risk stroke populations.

Despite preclinical promise, probiotic and prebiotic use in IS patients raises critical safety concerns. First, SIDS—characterized by lymphopenia, monocyte deactivation, and suppressed innate immunity within 24–72 h post‐ictus—substantially elevates the risk of bacteremia and systemic sepsis from live probiotic organisms, even nominally safe strains such as *Lactobacillus* and *Bifidobacterium* spp., particularly in patients with central venous catheters or severe stroke (NIHSS > 15). Second, dysphagia, affecting 30%–50% of acute stroke patients, poses a direct barrier to oral administration and introduces aspiration risk; enteral (nasogastric) delivery may be considered but adds procedural complexity. Third, concurrent broad‐spectrum antibiotic use for SAP substantially reduces probiotic colonization efficiency, potentially negating therapeutic benefit. Fourth, patients with severe hepatic impairment, short bowel syndrome, or prosthetic cardiac valves represent particularly high‐risk subgroups. In these populations, heat‐killed postbiotic preparations or purified prebiotic fibers may offer safer alternatives, retaining immunomodulatory and barrier‐protective properties without viable‐organism sepsis risk. These considerations underscore the need for rigorous patient stratification by immune status, careful route selection, and dedicated safety monitoring in any clinical trial of probiotic interventions in stroke populations.

### 7.2 FMT

FMT represents perhaps the most comprehensive candidate intervention, with the potential to simultaneously address multiple pathophysiological axes identified in this review: it may help restore SCFA‐producing taxa, reinforce gut barrier integrity, and rebalance the Treg/Th17 immune axis. FMT achieves direct microbiota ecosystem reconstruction with robust preclinical efficacy. In preclinical rodent models, the results are encouraging. In aged mice receiving MCAO, young FMT administered at 3 days post‐stroke significantly enhanced motor and cognitive recovery and reduced depression‐like behaviors independently of infarct volume (J. Lee et al. 2020). FMT was associated with reinforced gut integrity, improved barrier function, expanded Treg populations in both gut and brain, alongside suppressed pro‐inflammatory IL‐17 + γδT cells, pointing to immune‐mediated neuroprotective pathways. Whether this Treg expansion mechanism translates meaningfully to the human context—where Treg depletion and γδT/Th17 polarization have been described as drivers of post‐stroke neuroinflammatory amplification—remains an open question.

Similarly, promising translational signals come from type 2 diabetic AIS models wherein FMT from butyrate‐pretreated db/db mice was associated with smaller infarct volumes, lower neurological deficit scores, enhanced intestinal barrier integrity with improved ileal and colonic morphology, and preserved cerebral capillary glycocalyx structure with increased tight junction protein expression (J. Lee et al. 2020).

Furthermore, fecal microbiota from *P. distasonis*‐treated rats transferred protective effects in hyperuricemic stroke models, reducing neurological deficits and inflammation while stabilizing BBB integrity (H. Wei et al. 2024), a finding consistent with the notion that *P. distasonis* depletion may contribute to neuroinflammation in hyperuricemia‐associated stroke. Collectively, these findings suggest that FMT might be a potent microbiota‐restorative intervention across diverse stroke phenotypes. However, these preclinical findings are compelling; clinical evidence for FMT in IS remains at an extremely early stage. To our knowledge, no completed, peer‐reviewed RCT has specifically evaluated FMT as a therapeutic intervention in IS patients. A small number of case reports and pilot feasibility studies suggest that FMT is safe and tolerable in neurological patients, but efficacy data are absent. Considerable biological, regulatory, and logistical challenges—including donor selection, timing of administration, route of delivery, and standardization of endpoints—must be addressed before FMT can be considered a viable clinical option in stroke management. The compelling animal data should therefore be interpreted as hypothesis‐generating, warranting carefully designed phase I/II clinical trials.

The mechanistic transmissibility of stroke outcomes via FMT—demonstrated by the finding that both TMAO generation capacity and stroke severity are transmissible traits (Zhu et al. 2021)—provides some of the most persuasive evidence that microbiota composition may causally influence ischemic injury severity, and has led some researchers to position FMT as a logical therapeutic direction grounded in the causal microbiota–stroke relationship established throughout this review.

Translating FMT from preclinical stroke models to clinical practice requires careful attention to feasibility and safety, with infection risk being the major concern. FMT can transmit donor‐derived pathogens (including multidrug‐resistant organisms, MDROs). US FDA safety alerts (2019–2020) reported severe infections and deaths linked to ESBL‐producing *Escherichia coli* transmission via FMT in immunocompromised recipients, underscoring the potential for fatal outcomes with inadequate donor screening (DeFilipp et al. 2019; Nicholson et al. 2021). Stroke patients may be especially vulnerable because SIDS and acute gut‐barrier disruption can facilitate microbial translocation, while advanced age, hospitalization, and frequent antibiotic exposure increase MDRO colonization risk. In addition, post‐stroke dysphagia often limits oral/capsule delivery, making colonoscopic or enema administration more likely and adding procedure‐related risks in patients who are hemodynamically unstable or receiving antithrombotic therapy after reperfusion treatments. Therefore, FMT in IS should currently be regarded as investigational and restricted to rigorously monitored clinical trials with standardized donor screening (e.g., MDROs/ESBL, enteric pathogens, SARS‐CoV‐2, and HBV/HCV, HIV), explicit exclusion criteria (severe immunosuppression, active infection, and recent broad‐spectrum antibiotics), and prospective adverse‐event reporting. WMT may reduce transmission risk and improve standardization but requires dedicated safety validation in stroke populations.

### 7.3 Targeting Microbial Metabolites

Interventions targeting metabolites represent the most mechanistically precise therapeutic approach, allowing pathway‐specific correction of the key metabolic axes involved: SCFA deficiency (GPR41/43‐PI3K/Akt neuroprotective axis), tryptophan catabolism dysregulation (AhR‐Treg‐IL‐10 neuroimmune axis), bile acid signaling impairment (TGR5/PKA‐NLRP3 anti‐neuroinflammatory axis), and TMAO‐driven atherothrombosis (platelet activation and foam cell formation axis). Gut microbiota‐derived metabolites—SCFAs, indole derivatives, and bile acids—are increasingly recognized as critical regulators of post‐stroke recovery amenable to precision targeting. Sodium butyrate significantly reduced infarct volume and improved neurological deficits at 24 and 72 h post‐MCAO, with sustained functional improvements at 4 weeks (Z. Zhou et al. 2021). Mechanistically, butyrate could activate the GPR41/Gβγ/PI3K/Akt pathway, reducing neuronal apoptosis in ischemic penumbra regions through HDAC inhibition, suggesting a link between metabolite signaling and epigenetic neuroprotection. This GPR41/PI3K/Akt pathway is of particular interest given the butyrate deficiency and epigenetic dysregulation that have been documented in association with microglial pro‐inflammatory polarization and BBB compromise. These mechanistic insights from preclinical studies are promising, but it should be emphasized that direct butyrate supplementation in human stroke patients has not been formally evaluated in clinical trials. The bioavailability of exogenous butyrate, its capacity to cross the BBB at therapeutic concentrations, and its safety profile in the context of acute brain injury remain important unresolved questions. Indole‐3‐propionic acid (IPA), an AhR ligand, demonstrated significant therapeutic potential: serum IPA levels were markedly depleted in MCAO mice, while intragastric IPA administration substantially improved neurological deficits, reduced infarct volume, and reshaped dysbiotic microbiota by enriching Lactobacillus and Lachnospiraceae_NK4A136_group (Peesh et al. 2025). IPA was associated with enhanced tight junction protein expression, modulated Treg and Th17 cell activities, and ameliorated A1 neurotoxic astrogliosis through Treg‐mediated IL‐10 release, implicating the tryptophan‐kynurenine pathway as a potentially relevant mediator of both post‐stroke neuroinflammation and PSD. As with butyrate, the translation of IPA supplementation to human stroke therapy remains speculative at this stage; controlled human studies are lacking. Bile acid signaling further extends this paradigm: UDCA, a TGR5 agonist, significantly reduced infarct volumes and improved neurological outcomes through TGR5/PKA signaling, suppressing NLRP3 inflammasome‐mediated neuroinflammation (F. Zhang, Deng, et al. 2024). Notably, UDCA has a well‐established safety record in hepatobiliary disease, making it one of the more clinically tractable metabolite‐targeting candidates; however, dedicated clinical trials in stroke populations are still needed. The TGR5‐NLRP3 relationship observed in these studies is consistent with the bile acid depletion and NLRP3 inflammasome activation that have been documented as downstream consequences of post‐stroke gut dysbiosis, though causal directionality in humans has yet to be established. These metabolite‐targeted approaches offer potentially valuable precision therapeutic opportunities beyond whole microbiota modulation, though their translation from bench to bedside requires rigorous clinical evaluation.

Critically, TMAO reduction strategies—achievable through dietary phosphatidylcholine restriction, FMO3 inhibition, or gut microbial community restructuring away from cutC/cutD‐expressing taxa—have been proposed as potential interventions targeting the atherothrombotic risk mechanisms established as associated with stroke severity and recurrence (Zhu et al. 2021; Haghikia et al. 2018). This positions TMAO‐targeted metabolite interventions as potentially more suitable for primary and secondary stroke prevention, whereas SCFA/tryptophan/bile acid supplementation strategies appear better suited as acute and rehabilitative phase interventions.

### 7.4 TCM and Acupuncture

TCM compounds and acupuncture have been reported to exert their microbiota‐mediated neuroprotective effects that might operate through two of the mechanistic axes established in earlier sections: (i) suppression of the LPS‐TLR4/MyD88/NF‐κB neuroinflammatory cascade implicated in post‐stroke BBB disruption and microglial priming, and (ii) shifts in tryptophan metabolism toward indole derivatives and away from the pro‐inflammatory kynurenine pathway that has been linked to PSCI and PSD. TCM compounds and acupuncture modulate gut dysbiosis through integrated pharmacological and neuroimmunological mechanisms. Notoginsenoside R1 (NG‐R1) treatment significantly reduced gut dysbiosis, intestinal inflammation, and systemic inflammation in MCAO/reperfusion rats through suppression of the TLR4/MyD88/NF‐κB signaling pathway, restoring BBB structure (S. Zhang, Chen, et al. 2024). This TLR4/MyD88/NF‐κB suppression directly reverses the LPS‐mediated endothelial dysfunction and neuroinflammatory priming cascade established as the primary driver of intestinal epithelium‐originating stroke pathophysiology. Notably, FMT from NG‐R1‐treated rats conferred similar protective effects, supporting microbiota‐dependent mediation. Huang‐Qi‐Long‐Dan‐Gen particles (HQLDG) multi‐omics analyses revealed enhanced gut microbiota composition, augmented tryptophan metabolism, and suppression of Th17/IL‐17 signaling (Wu et al. 2024); microbiota depletion impaired HQLDG therapeutic efficacy, while fecal microbiota from HQLDG‐treated mice improved transient MCAO outcomes, confirming that microbiota mediation is at least partly required for its effects. These data suggest that HQLDG may operate through the tryptophan metabolism axis—specifically the indole/AhR branch that has been associated with Treg differentiation and suppression of pathogenic Th17 responses. Buqi‐Huoxue‐Tongnao Decoction (BHTD) increased gut microbiota‐derived indole lactic acid (ILA) via tryptophan upregulation, which was associated with attenuated IS injury; ILA supplementation alone replicated these benefits, pointing to the tryptophan‐indole metabolic axis as a plausible operative mechanism (Y. Liu et al. 2024). HLJDD was shown to alleviate diabetic IS in association with restoration of gut microbiota‐derived SCFAs and inhibition of TLR4/NF‐κB signaling, with pseudo‐germ‐free models confirming microbiota dependency (C. Chen et al. 2025). This dual mechanism—SCFA restoration and TLR4/NF‐κB suppression—simultaneously could address two of the four convergent pathological features of comorbidity‐encoded dysbiotic pre‐conditioning. Additional compounds including berberine and baicalein (Song et al. 2024) demonstrate broadly convergent microbiota‐modulating and neuroprotective capacities. Electroacupuncture combined with induced pluripotent stem cell‐derived extracellular vesicles (iPSC‐EVs) demonstrated synergistic neuroprotection and microbiota modulation, improving neurological function, reducing brain and intestinal injury, markedly altering microbiota composition with increased Akkermansia abundance, and regulating gut immunity through reduced pro‐inflammatory IL‐17 and elevated anti‐inflammatory IL‐10 in brain and colon tissues (Q. Zhang, Deng, et al. 2023). While acupuncture has been evaluated in stroke rehabilitation trials, these studies have not incorporated microbiome endpoints; therefore, the microbiota‐mediated mechanisms described here remain hypothetical in the human context and require dedicated clinical investigation.

### 7.5 Emerging Therapeutic Strategies

Emerging therapeutic modalities—including neuromodulation, social interventions, and nanomaterial‐based approaches—appear to converge on shared mechanistic nodes established throughout this review, with their therapeutic potential in stroke at least partly attributable to these shared pathways rather than to wholly independent mechanisms. Multiple innovative modalities demonstrate therapeutic potential through gut microbiota modulation. Neuromodulation techniques include VNS, which alleviated motor deficits, GI dysfunction, and neuroinflammation in MCAO/reperfusion rats by stabilizing mast cells, reducing degranulation, mitigating intestinal and BBB damage, regulating gut microbiota composition, and reducing LPS leakage and systemic inflammation (Y. Wang, Tan, et al. 2024). VNS operates through the vagal‐cholinergic anti‐inflammatory axis established in Section 2.2 as a major neural pathway of gut‐brain communication in stroke—specifically, by restoring the cholinergic anti‐inflammatory reflex that is impaired by stroke‐induced dysbiosis‐driven vagal dysfunction. These effects are consistent with the involvement of the vagal‐cholinergic anti‐inflammatory pathway, whereby stroke‐induced dysbiosis may impair vagal function and cholinergic anti‐inflammatory signaling. Transcranial direct current stimulation (tDCS) conferred neuroprotection in association with bile acid metabolism modulation, reducing apical sodium‐dependent bile acid transporter (ASBT) expression and 3‐oxoLCA levels, decreasing infarct volume and neuronal death (Kong et al. 2023). This bile acid‐related mechanism may involve the FXR/TGR5 signaling pathways linked to BBB integrity and microglial homeostasis. Social interventions such as pair‐housing (cohabitation with healthy isosexual partners) significantly improved anxiety and depression‐like behaviors in PSD mice, increased gut microbiota richness and diversity, improved gut barrier disorders and inflammation, decreased CD8 + lymphocytes and pro‐inflammatory cytokines (TNF‐α, IL‐1β, and IL‐6) while upregulating anti‐inflammatory IL‐10, and reduced activated microglia while increasing hippocampal Nissl bodies (S. T. Jiang et al. 2024). These observations are consistent with a pathway in which increased microbiota diversity promotes SCFA restoration, which in turn supports BBB integrity and limits microglial activation—processes that have been implicated in PSCI pathogenesis. Manganese‐ferric Prussian blue nanozymes substantially reduced infarct volume, improved neurological function, restored gut microbiota balance, and increased SCFA levels, mechanistically operating through TLR4/NF‐kB pathway suppressio—again targeting the LPS‐TLR4‐NF‐κB neuroinflammatory axis as the convergent mechanistic node.

Collectively, these diverse therapeutic modalities share several common features: restoration of SCFA‐producing taxa, reinforcement of intestinal barrier integrity, modulation of peripheral and central immune responses, and metabolite‐mediated signaling. Taken together, these preclinical data support continued investigation of microbiota‐targeted approaches as part of a broader framework for stroke rehabilitation, while the absence of robust clinical trial data remains a critical limitation.

### 7.6 Limitations of Current Therapeutic Evidence

Despite mechanistic coherence and promising preclinical efficacy, current therapeutic evidence targeting the gut–brain axis in stroke is constrained by substantial methodological limitations across all intervention categories. Probiotic and prebiotic trials remain limited by small sample sizes (*n* < 100), short durations, heterogeneous populations, and reliance on surrogate biomarkers rather than hard clinical endpoints, with dose standardization and strain selection inadequately addressed. FMT, despite compelling preclinical data, including landmark aged‐to‐young microbiota transfer experiments, has no established human trials in stroke populations, with critical questions surrounding patient safety, administration timing, donor selection, and delivery route remaining unresolved. TCM interventions suffer from single‐center designs, limited blinding, non‐standardized herbal preparations, and unclear attribution of microbiota‐modulating effects to specific constituents; herb–drug interactions in polypharmacy stroke patients and restriction to Chinese populations further limit applicability. Metabolite‐targeted strategies, including SCFA supplementation and TMAO pathway inhibition, lack defined dosing regimens and therapeutic windows in humans, with DMB remaining unevaluated in clinical trials and PAGln‐targeted approaches at the proof‐of‐concept stage. Collectively, over‐reliance on animal models, absence of adequately powered randomized controlled trials, and insufficient patient stratification represent critical barriers to clinical translation.

### 7.7 Mechanistic Convergence and Therapeutic Logic: Mapping Therapies to the Three‐Phase Stroke Model

The therapeutic landscape can be organized around a three‐phase pathophysiological model, providing phase‐specific mechanistic context for the interventions reviewed above. Phase I (pre‐stroke priming): Primary prevention. TMAO‐driven atherothrombosis (Zhu et al. 2021), PAGln‐mediated NET formation (Nemet et al. 2020; M. Wei et al. 2025), and LPS–TLR4 endothelial dysfunction (Koszewicz et al. 2021) represent potentially important upstream targets supported by causal inference in preclinical models and by observational clinical data. Dietary substrate restriction, TMA‐lyase suppression, and SCFA‐producer restoration have been proposed as means of addressing these pathways prior to ischemic onset and could inform a microbiota‐aware primary prevention strategy. Phase II (Acute microbial–neural crosstalk): Neuroprotection. The LPS–TLR4–NF‐κB cascade may function as a dominant acute amplification loop (S. Zhang, Chen, et al. 2024). Intestinal TLR4 inhibition, butyrate supplementation (GPR41/PI3K/Akt) (Z. Zhou et al. 2021), FMT‐mediated Treg/Th17 rebalancing (J. Lee et al. 2020), and VNS (Y. Wang, Tan, et al. 2024) each target this loop at mechanistically distinct nodes, with the potential to attenuate infarct expansion and secondary neuroinflammation. Phase III (recovery‐phase reprogramming): Rehabilitation. Persistent SCFA deficiency (J. Lee et al. 2020), tryptophan–kynurenine dysregulation (Peesh et al. 2025), and Proteobacteria‐driven neuroinflammation have been associated with PSCI and PSD. AhR‐directed indole supplementation, TGR5/PKA–NLRP3 modulation (F. Zhang, Deng, et al. 2024), and microbiota‐targeted TCM formulations (Q. Zhang, Deng, et al. 2023; Wu et al. 2024) address these chronically dysregulated axes. Across all three phases, therapeutic strategies tend to converge on five shared nodes: SCFA restoration, gut barrier reinforcement, Treg/Th17 rebalancing, GPR41/43–FXR/TGR5–AhR metabolite signaling, and LPS–TLR4–NF‐κB suppression. This mechanistic convergence supports the view that microbiota‐targeted therapy warrants consideration as a causally grounded component of precision stroke care, though this perspective must be tempered by the current absence of clinical trial validation.

## 8 Conclusion

**FIGURE 2:**
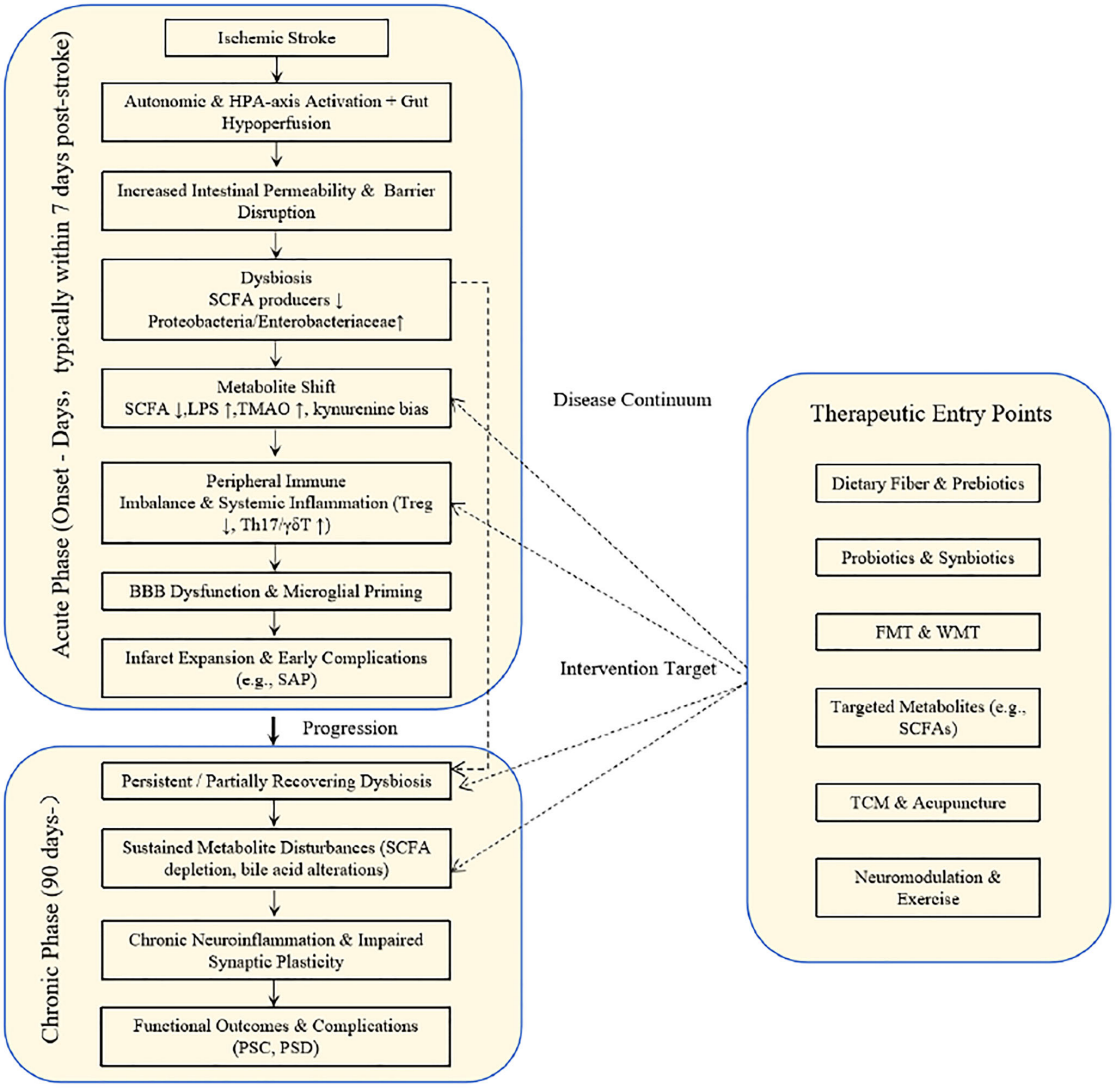
Mechanisms of the microbiota–gut–brain axis across acute vs. chronic phases of ischemic stroke (summary schematic). Acute phase (Onset–days, typically within 7 days post‐stroke): stroke → autonomic/HPA‐axis activation + gut hypoperfusion → increased intestinal permeability and barrier disruption → dysbiosis (↓SCFA‐producing taxa; ↑Proteobacteria/Enterobacteriaceae) → metabolite shift (↓SCFAs; ↑LPS; ↑TMAO; kynurenine bias) → peripheral immune imbalance (↓Treg; ↑Th17/γδT) + systemic inflammation → BBB dysfunction and microglial priming → infarct expansion and early complications (e.g., SAP, GI dysfunction). Chronic phase (90 days): persistent/partially recovering dysbiosis + sustained metabolite disturbances (SCFA depletion; bile acid/tryptophan pathway alterations) → chronic neuroinflammation and impaired synaptic plasticity → functional outcomes and complications (PSCI and PSD), with recovery trajectories influenced by host factors (age, sex, and comorbidities) and rehabilitation‐related exposures (diet, medications, and feeding route).

The gut microbiota plays a central role in IS pathophysiology, with its modulation representing a critical target for improving stroke outcomes. Multiple therapeutic strategies—including probiotics, prebiotics, synbiotics, FMT, targeted metabolite supplementation, TCM with acupuncture, and emerging neuromodulation and social interventions—modulate the MGBA through distinct mechanisms, with robust efficacy demonstrated in preclinical experimental models. However, it is critical to acknowledge that human clinical evidence for most of these interventions in stroke remains limited: probiotics and dietary interventions have the most preliminary clinical support, whereas FMT, targeted metabolite supplementation, TCM‐mediated microbiota modulation, and neuromodulation approaches are supported primarily or exclusively by animal data. The gap between preclinical promise and clinical validation represents a major challenge for the field. Future investigations should prioritize rigorous randomized controlled trials with appropriate sample sizes, standardized microbiome outcome measures, and mechanistic biomarker tracking to establish microbiota‐targeted strategies as evidence‐based adjunctive stroke therapies. Notably, host factors including sex, age, and comorbidities significantly influence therapeutic efficacy, highlighting the importance of personalized precision treatment. Beyond efficacy considerations, clinical safety represents an equally critical dimension: stroke‐induced immunodepression, dysphagia, antibiotic exposure, and multimorbidity collectively elevate the risk of adverse events associated with live probiotic administration (bacteremia and sepsis) and FMT (donor‐pathogen transmission and procedural complications), underscoring the imperative for rigorous patient selection, safety stratification, and adverse event monitoring in future clinical trials of microbiota‐targeted stroke therapies. Future investigations should prioritize precision microbiome medicine approaches with individual microbiota profiling, sex‐specific considerations, and comorbidity‐tailored interventions while exploring synergistic combinations of multiple therapeutic strategies to provide more effective microbiota‐targeted treatments for IS patients (Figure 2).

## 9 Limitations and Inconsistencies

Several limitations constrain the conclusions drawn from existing evidence. The predominance of cross‐sectional designs prevents causal or directional inference—gut dysbiosis observed post‐stroke may reflect neurological injury and immobility rather than a predisposing factor. Microbial signatures vary substantially across cohorts due to geographic, dietary, and methodological heterogeneity, and no consistent stroke‐associated microbial profile has emerged. Preclinical evidence is similarly limited: the near‐universal use of young, inbred, comorbidity‐free rodents in MCAO models poorly reflects the clinical complexity of human stroke. Human intervention trials, meanwhile, remain underpowered and reliant on surrogate endpoints, yielding insufficient evidence to support microbiota‐targeted therapies in routine practice. Confounding by antibiotics, immobility, and comorbid conditions further complicates clinical microbiota research. Progress will require prospective cohorts with pre‐stroke sampling, shotgun metagenomic approaches integrated with metabolomics, sex‐balanced and comorbid preclinical models, and adequately powered randomized trials with hard cerebrovascular endpoints. Until such evidence accumulates, the gut–brain–vascular axis remains a mechanistically compelling but clinically unverified framework.

## Author Contributions

**Ruihui Weng**: conceptualization, investigation, methodology, validation, writing – original draft. **Gaolei Dong**: conceptualization, data curation. **Jianxin Tang**: conceptualization, methodology, software, data curation. **Yu Zhao**: conceptualization, investigation, methodology, validation, formal analysis, software, data curation, supervision, writing – review and editing.

## Funding

The research work was supported by Shenzhen Clinical Research Center for Neurological Diseases (LCYSSQ20220823091204009, SCRCND202501), Shenzhen High‐level Hospital Construction Fund.

## Ethics Statement

This study is a literature‐based narrative review and did not involve the collection, use, or analysis of data from human participants or animals. Therefore, ethical approval and informed consent were not required for this work and are not applicable.

## Consent

The authors have nothing to report.

## Conflicts of Interest

The authors declare no conflicts of interest.

## Data Availability

The data that support the findings of this study are available from the corresponding author upon reasonable request.
